# An Evaluation of Malaysian Regulatory Process for New Active Substances Approved in 2017 Using the OpERA Methodology

**DOI:** 10.1007/s43441-020-00140-4

**Published:** 2020-03-28

**Authors:** Noraisyah Mohd Sani, Neil McAuslane, Siti Hidayah Kasbon, Rosilawati Ahmad, Faridah Aryani Md. Yusof, Prisha Patel

**Affiliations:** 1 National Pharmaceutical Regulatory Agency, Lot 36, Jalan Universiti, 46200 Petaling Jaya, Selangor Malaysia; 2 Centre for Innovation in Regulatory Science (CIRS), 160 Blackfriars Road, London, SE18EZ UK

**Keywords:** National Pharmaceutical Regulatory Agency (NPRA), Optimising Efficiencies in Regulatory Agencies (OpERA), Regulatory strengthening, Benchmarking

## Abstract

**Introduction:**

The National Pharmaceutical Regulatory Agency (NPRA) embarked on a regulatory-strengthening program and is evaluating its processes. Optimising Efficiencies in Regulatory Agencies (OpERA) is a regulatory-strengthening program that provides benchmarking data that can define performance targets and focus performance improvement. The objective of this study was to use OpERA methodology to determine where time is spent in the NPRA approval process and to form a baseline to measure the performance improvements.

**Methods:**

The OpERA tool was used to collect specific milestone data that identify time periods, review stages, and data points for new active substances and biosimilars approved by NPRA in 2017.

**Results:**

In 2017, 25 new active substances and 1 biosimilar were approved by NPRA in a median of 515 days, representing both agency and applicant time. The median time between dossier receipt and the initiation of NPRA scientific assessment was 135 days, but there was a wide variation in queuing time. The median total assessment time was 279 days (agency and applicant timing). NPRA took a median of 166 days; applicants took a median of 131 days to respond to deficiency questions, with up to 6 cycles of review required for approval and 65% of applications requiring 4–5 cycles to provide satisfactory responses.

**Conclusions:**

As a result of these data, NPRA proposes three improvements: target start for scientific assessment 100 days after file acceptance, a maximum of 5 review cycles, and applicant response time limited to 6 months. These results will serve as a baseline for further assessment.

## Introduction

As the development and availability of new medicines become increasingly important for healthcare systems, regulatory agencies are strengthening their review of these therapeutics in order to fulfil their mandate to protect and promote public health [1, 2]. This strengthening along with the building of regulatory technical capacity facilitates regulatory agencies in their mission to enable patient access to good-quality, safe, and effective medicines in a timely manner.

To maximize the use of limited resources, many agencies have evolved their processes to employ risk-based approaches, including reliance on prior reviews by reference agencies while also taking into consideration benefit-risk decisions based on local standards of care. These reliance approaches are supported by international regulatory convergence and alignment around guidelines such as those of the International Conference on Harmonisation of Technical Requirements for Registration of Pharmaceuticals for Human Use (ICH) and the Association of Southeast Asian Nations (ASEAN) and are underpinned by good review practices [3–6].

Implementation of these approaches faces a number of legal, political, methodological, and organizational challenges; however, the challenges can be mitigated by ensuring that agencies have the relevant capabilities, decision-making frameworks, and practices and are also measuring their own performance in a systematic and structured way.

The measurement of regulatory review performance should be documented and tracked so as to identify where time is spent, thus ensuring the efficiency of a review process as it evolves. Hence, the need for agencies to proactively and consistently measure their performance against stated target times is one of the World Health Organization (WHO) global benchmarking tool indicators [7].

### Malaysia

Classified by the World Bank as an upper middle-income economy, Malaysia has a multi-cultural, multi-ethnic population of approximately 32 million people. Healthcare spending accounts for 4.25% of the country’s $933 billion USD annual gross domestic product [8] and in 2017, the government named healthcare as one of the country’s twelve National Key Economic Areas and dedicated 10% of its annual budget to healthcare spending [9]. The current value of the pharmaceutical market in Malaysia is approximately $3 billion USD, with an annual 10% growth forecasted [9].

### The National Pharmaceutical Regulatory Agency

Instituted in 1978, the National Pharmaceutical Regulatory Agency (NPRA) formerly known as the National Pharmaceutical Control Bureau (NPCB) is responsible for ensuring the quality, safety, and efficacy of pharmaceuticals in Malaysia. The agency was accepted as the 30^th^ member of the World Health Organization (WHO) Program for International Drug Monitoring in 1990 and in 1996; the WHO designated NPRA as a WHO Collaborating Centre for Regulatory Control of Pharmaceuticals. In addition, as part of the ASEAN Technical Cooperation among Developing Countries (ASEAN TCDC) program, NPRA was named a Regional Training Centre for Quality Control of Pharmaceuticals, and the agency has conducted regulatory training workshops and seminars for representatives from many countries, including Bangladesh, Mongolia, Nigeria, Sri Lanka, and Tanzania. NPRA quality management certifications include the MS ISO 9001:2015 as well as the MS ISO 17025:2005, and in 2002, the NPRA became a member of the Pharmaceutical Inspection Cooperation Scheme (PIC/S), indicating that Malaysian good manufacturing practice is aligned with international standards [10]. NPRA is also recognized internationally as an Organisation for Economic Cooperation and Development (OECD) member fully compliant with the Good Laboratory Practice (GLP) Mutual Acceptance Data (MAD) System since 2013 [11] and is an ICH observer since 2018 [12].

NPRA has embarked on a regulatory-strengthening and performance improvement program and this is in line with the theme for the 2018 National Regulatory Conference, Regulatory Excellence; The New Normal. As part of this program, the agency is evaluating its processes and systems to achieve its mission to safeguard the nation’s health through scientific excellence in the regulatory control of medicines and cosmetics as well as to achieve its vision to be an internationally renowned regulatory agency [11].

The Centre for Innovation in Regulatory Science (CIRS) is a neutral international forum for 21 pharmaceutical company members and representatives from academia, regulatory and health technology assessment agencies, and other healthcare stakeholders to advance policies and processes in the regulation and reimbursement of medicines through research, workshops, publications, and advocacy. CIRS has developed a unique regulatory-strengthening program called OpERA: Optimising Efficiencies in Regulatory Agencies to aid NPRA and other agencies in these important goals. OpERA is a multi-year project initiated by CIRS in 2013 based on requests from regulatory agencies [13]. Program objectives are to (1) provide benchmarking data that can be used to define performance targets and focus ongoing performance improvement initiatives, (2) accurately compare the processes used in the review of new medicine marketing authorizations, (3) encourage the sharing of information on common practices in order to learn from others’ experiences, and (4) encourage systematic measuring of the processes that occur during the review of new medicine marketing authorizations [14].

The program is designed to support the metrics/information needs of mature and maturing authorities and has the key goals of partnering with regulatory agencies to conduct ongoing, systematic assessments of the processes that occur during the review of a marketing authorization. It also provides the tools to help integrate a practice of collecting ongoing information to encourage consistent internal tracking and assessments. For agencies, the envisioned outcome of participation is the receipt of factual results that can be used to help better convey their mission and needs to policy-makers and other stakeholders as well as to continuously monitor their performance for purposes of improvement of timelines and quality of processes.

**Figure 1.**
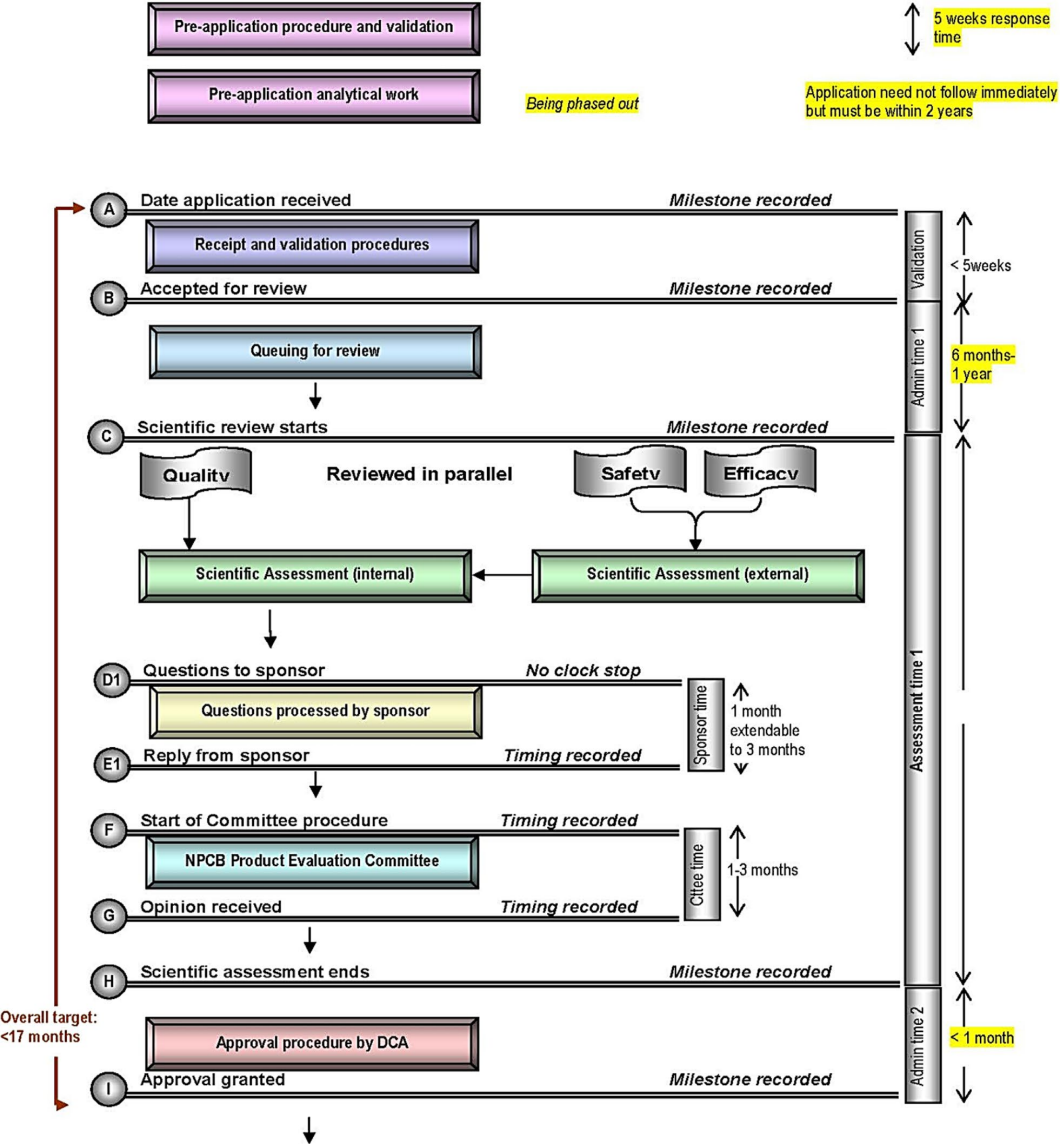
NPRA Review Process.

With this background, NPRA agreed to join the OpERA program and provided both qualitative and quantitative information regarding its approval process, as described in the following narrative and illustrated in Fig. 1.

**NPRA review process**

For pre-marketing approval, documentation for new chemical entities (NCEs) and biologics submissions must adhere to the ASEAN Common Technical Dossier (ACTD) format that is submitted via an online system, known as Quest3. In addition to the adherence to the ACTD dossier formatting and the NPRA Drug Registration Guidance Document [15], the requirements for registration of NCEs and biologics in Malaysia are also consistent with the guidance established by WHO, ICH, and other international guidelines published by European Medicines Agency (EMA) or the US Food and Drug Administration (US FDA). Once the application is received via the Quest3 system, the data will be validated for completeness of the dossier. The file will be accepted for review if all essential data are provided.

For new chemical entities and biologics, the target timeline is set at 245 working days for the standard registration pathway, indicated as Path II. Products using this registration pathway must be registered in at least one country that follows harmonized regulatory requirements.

Registration timelines are shortened to 120 working days for certain products, which are assigned priority review or fast track status, indicated as Path I. Products may be designated as Path I if they are potentially lifesaving or are intended for an unmet medical need with no treatment options locally available, for example, a rare disease or oncology indication, or if they address a threat to public health. In addition, the first biosimilar product or the first locally manufactured biosimilar product may be designated as Path I where no biosimilar product has been registered by the Drug Control Authority at the time of granting priority review.

The comprehensive regulatory mechanisms encompass internal review on the quality, efficacy, and safety characteristics. The views and opinions from subject matter experts are also sought, particularly on the clinical studies and practices. NPRA will correspond with the applicant to request for further documents or information. When review is complete, the final evaluation report will be presented in the NPRA evaluation committee meeting. Finally, it will be presented to the Drug Control Authority meeting for final decision as to whether to grant marketing authorization or to reject the application.

The objective of this study was to use OpERA methodology to provide NPRA with a breakdown of where the time is spent in their approval process and to form a baseline against which future performance improvements can be measured. This article reports on a selection of analysis from the first year of OpERA data collection and provides the baseline for NPRA to evaluate ongoing changes made to strengthen its regulatory review process to meet its vision and goals.

## Methods

This article does not contain any studies with human or animal subjects performed by any of the authors.

The OpERA methodology is based on the collection of specific milestones that identify time periods, review stages, and data points, which can be compared across regulatory agencies, notwithstanding the considerable differences between the individual regulatory procedures. The milestone dates set out as data points in Table 1 were collected for each application in the study as applicable to the particular process. In addition, qualitative data were requested for each product in order to characterize the application. These data included applicant name; whether the application was from a multinational or local company; the compound type; that is, new chemical entity, biological, or vaccine, the generic name or compound code; whether the compound was a WHO pre-qualified generic or vaccine; the trade name; the review type; that is, verification, abridged, or full; the general therapeutic class identified by ATC code; and whether it was a priority review. Applicant names, generic name/compound codes, and trade names could be masked for confidentiality.

**Table 1.** Datasets Collected Through the OpERA Program.

Key Milestone Date	Time
1a. Receipt of the dossier	Dossier validation time
1b. Acceptance to file	Time to acceptance of the dossier
2a. Start of primary scientific assessment	Primary scientific assessment
2b. Completion of primary scientific assessment	
3a. Primary assessment deficiency letter sent to applicant (if applicable)	Clock stop/Applicant time
3b. Response from applicant (if applicable)	
4. Secondary assessment following deficiency letter response (if applicable)	Secondary scientific assessment time
5. Succeeding Advisory Committee review (if applicable)	Advisory Committee time
6. Completion of Scientific Assessment	When all assessment activities are completed
7. Marketing Authorization granted\ rejected	Date granted/rejected

### Collection of Data from NPRA

Following a CIRS-conducted WebEx training session, NPRA supplied datasets for new active substances (NASs) consisting of new chemical entities and biologics approved between January 1, 2017, and December 31, 2017, using a CIRS-supplied Microsoft Excel spreadsheet. CIRS then validated the integrity of milestone date information and evaluated the missing data. A draft analysis was prepared and presented in an in-person visit to NPRA to ensure agency understanding of the dataset and consistency of data provision.

### Time Periods

All time periods were measured in calendar days for this study. Using the OpERA tool, elapsed time during a review was categorized under the following headings:

- *Dossier validation:* The time between the date stamped on receipt of the dossier and the date of sending the acceptance- (or refusal-) to-file letter
- *Queue time*: Time between accepting the dossier for review and starting the review
- *Scientific assessment time:* Time spent between the date of the start of the scientific assessment to the date of completion of all scientific assessments
- *Agency scientific assessment time:* Amount of time spent between the date of the start of the scientific assessment to the date of the completion of all scientific assessments, minus the time the applicant needs to prepare responses to questions or any additional information is provided
- *Applicant time:* The time during which the review timing clock is stopped during the review while the authority awaits additional data requested from the applicant
- *Authorization time:* The time from completion of all scientific assessments to the authorization/license date that allow legal marketing
- *Overall approval time:* The time between the date stamped on the receipt of dossier when received by authority and the date on the document (authorization/license) that allows legal marketing

### Analysis: Review Stages and Data Points

Three main stages of the approval processes were analyzed using the milestones dates for (1) time from receipt of data to start of scientific assessment, (2) scientific assessment (including both agency assessment and applicant response), and (3) authorization of the product. These data were then described statistically using medians and percentiles, in particular, the 5th, 25th, 50th, 75th, and 95th percentiles, to facilitate the understanding of the variation around the median.

## Results

Twenty-five new active substances (NASs) and one biosimilar were approved by the NPRA in 2017, of which 14 (54%) were new chemical entities and 12 (46%) were biologics. Data analyzed for each product included whether the provided review was path I (priority medicines with expedited approval) or path II (standard review). Of products approved in 2017, 5 (19%) had a path I designation (Table 2).

**Table 2.** Characteristics of Products Approved by NPRA in 2017.

Characteristics	2017	Chemical	Biologic
No of approved: 25 new active substances and 1 biosimilar	26	14	12^a^
Type of review			
Full	26	14	12
Priority review			
Yes	5	4	1
No	21		
Assessment route			
Path I	5	4	1
Path II	21		
Applicant type			
Multinational	20	12	8
Local	6	2	4
Therapy area			
Alimentary and metabolism	2		2
Anti-cancer and immunomodulators	9	2	7
Anti-diabetic	1	1	
Anti-infectives	4	4	
Cardiovascular	4	2	2
Genitourinary and sex hormones	1	1	
Ophthalmologicals	1	1	
Other	1		1
Respiratory	3	3	

^a^One biologic was a vaccine and one was a biosimilar.

**Figure 2.**
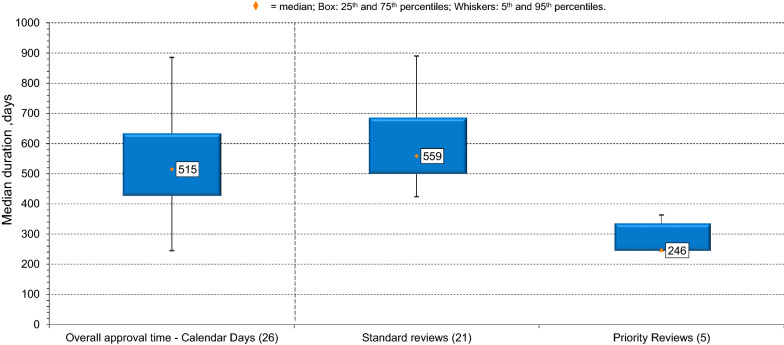
Overall Approval Time by Review Pathway for 25 NASs and One Biosimilar Approved by NPRA in 2017. (*n*) = Number of Applications.

Figure 2 shows the median approval time and variation for the 25 NASs and one biosimilar approved by NPRA in 2017 both overall and broken down by the standard and priority review routes. NPRA timeline for standard review of new chemical entities and biologics is 365 days and 180 days for priority review [15]. However, these targets are for agency timing only and the timing shown in Fig. 1 represents both agency and applicant time.

Of the five products that underwent a priority review, three were anti-infectives, one was an anti-cancer product, and one was classified as ATC other. Median review time for these five products was 246 days, compared with 559 for products undergoing a standard review.

### Breakdown of Time Spent in the Approval Process

**Figure 3.**
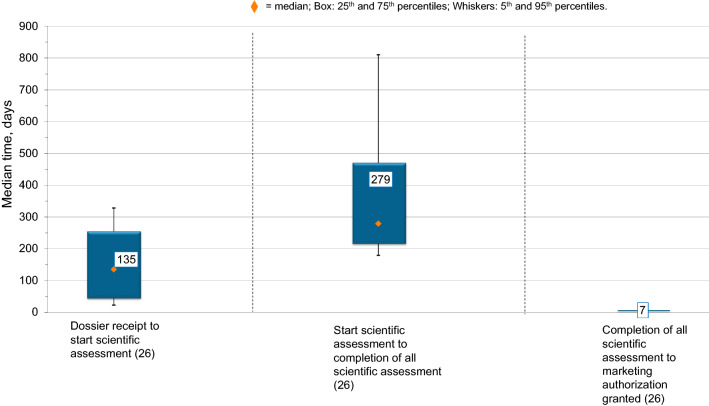
Three Main Components of the Approval Process for 25 NASs and One Biosimilar Approved by NPRA in 2017. (*n*) = Number of Applications.

Figure 3 shows the median time and variation that the products approved in 2017 spent in the three main stages of the approval process: dossier validation and queue time, scientific assessment, and authorization time. Comparing the three main stages of the NPRA approval process for medicines approved in 2017, the analysis shows that the agency spends the majority of its time in the scientific assessment phase of the process compared with the other two components. However, both the scientific assessment phase, which includes both agency and company time, and the time from dosser receipt to start of scientific assessment have wide variations; the variation from when the dossier was received by the agency to when it was picked up by the reviewers for scientific assessment ranged from 46 to 254 days (25–75 percentile) and the scientific assessment time, which included both agency and applicant time, ranged from 217 to 470 days. The median timing for the final stage of the review from the completion of all scientific assessments to marketing authorization was 7 days, which was consistent (Fig. 3).

### Breaking Down the First Component: Timing from Dossier Receipt to Start of Scientific Assessment

The two components of time from dossier receipt to start of scientific assessment are the validation stage (timing from submission to the agency until agency acceptance of the file), which was a median of 40 days in 2017 (variation, 23–77 days), and queue time (timing from agency acceptance until beginning of scientific assessment), which was a median of 105 days (variation, 19–171 days). As previously stated, the variation for the total of these two components was 46 to 254 days.

### Breaking Down the Second Component: Company and Agency Timing in the Scientific Assessment

**Figure 4.**
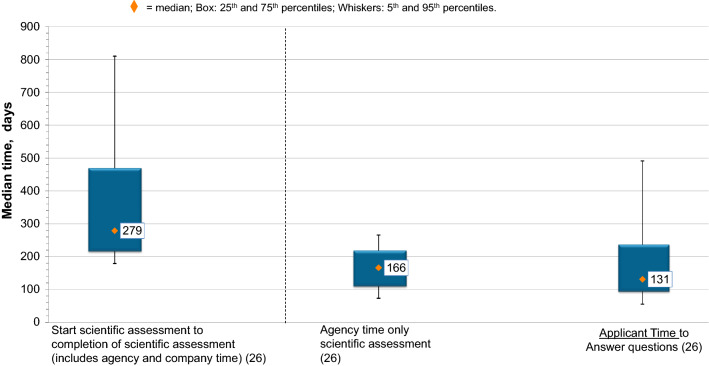
Agency and Applicant Scientific Assessment Time for 25 NASs and One Biosimilar Approved by NPRA in 2017. (*n*) = Number of Applications.

The total time taken by agencies to undertake the assessment and the companies to respond to the agency questions was similar, a median of 166 days versus 131 days, respectively. The variation around the median was also similar: variation, 25th–75th percentile, 110 to 220 days) and applicant timing to answer agency questions, variation, 94 to 238 days (Fig. 4).

Agency scientific assessment time was broken down further into primary scientific assessment, the time taken for the agency to review the dossier prior to sending out deficiency questions to the applicant followed by any subsequent time taken by the agency to review the answers to the agency’s questions. This process may be repeated for a number of review cycles. For NPRA, the median time for the primary scientific assessment for the 25 NASs and one biosimilar approved in 2017 was 50 days, resulting in approximately 29% of total agency scientific assessment time being spent in the primary review cycle.

The median time for applicants to provide primary responses to agency questions was 41 days (29% of total applicant response time). For this cohort of products, the completion of scientific assessment took two cycles for one product, three cycles for three products, four cycles for nine products, five cycles for eight products, and six cycles for five products.

## Discussion

Designated as a WHO Collaborating Centre for Regulatory Control of Pharmaceuticals and ASEAN TCDC Regional Training Centre for Quality Control of Pharmaceuticals, NPRA has made continual progress in strengthening its review process [10]. The primary reason the agency undertook this study was to identify areas for further improvement and to enable implementation of change. In addition, NPRA is planning to embed ongoing monitoring and measurement of its processes to provide an understanding of where time is being spent and to focus on improvement in its use of resources. Finally, NPRA aims to benchmark its performance against other established agencies; these goals are all aligned with the objectives of the OpERA program [13, 14].

### Study Confounders

It should be recognized that the overall results shown here may not reflect the true efficiency of the agency for several reasons. First, at the time of the study, the agency was undergoing a migration of its online dossier submission system, known as Quest3 to an improved version, Quest3+, resulting in various discrepancies in procedures. Second, as the system was not functioning at its full capacity, some of the applications were submitted manually in hard copy and correspondence was conducted via e-mail. The NPRA believes that some efficiency issues resulting from this system change may have impacted timelines during this period.

In this study, 25 NASs and 1 biosimilar were approved by the NPRA in 2017 in a median of 515 days. Although this gives an idea of the overall time spent in the approval process, it does not accurately identify in which of the components of the approval process, this time was spent and whether those components included agency and applicant time. Regulatory agencies must fully understand the breakdown of this timing to determine areas for efficiency improvement.

### Study Results Versus NPRA Goals

The current timeline established by NPRA to register new chemical entities and biologics is within 365 days, not including the time taken by the applicant to respond to agency queries [15]. All products approved in 2017 met this registration timeline. However, a breakdown of timing for the components of the review process is needed for full understanding, specifically, the time between dossier receipt and the initiation of NPRA scientific assessment and the agency and applicant timing within the assessment. Here, it is important to note that applicant timing may be influenced by agency performance and vice versa.

### Queuing Time Variation and Proposed Solutions

The median time between dossier receipt and the initiation of NPRA scientific assessment was 135 days across all products, with the validation stage being just over a month, followed by approximately 3 months queuing time. The wide variation around queuing time may be the result of differences across review divisions, with different internal and external resource constraints; however, in evaluating these data, NPRA believes that an agency target timeline could be established for the initiation of scientific assessment within 100 days after a file has been accepted for full review.

### Applicant Response Time and Number of Review Cycles and Proposed Solutions

Study results indicate a median of 166 days was required for agency review and as many as 6 cycles of review for approval. Applicants took an overall median of 131 days to respond to questions in 2017, which was nearly the same as the median amount of time required for scientific assessment by the agency. Moreover, 17 of 26 applications (65%) took between 4 and 5 cycles of review to fully respond to dossier deficiency questions.

The number of required cycles for review is a key area of concern for NPRA. Further study would be required to determine if this issue is caused by (1) review procedures that include a lack of agency consolidation of review questions, (2) insufficient applicant responses to agency questions, or (3) unsatisfactory responses by the applicant to agency communications regarding overall dossier quality. In reviewing these study results, NPRA believes that correspondence with the applicant could be limited to a maximum of 5 cycles in the newly upgraded Quest3+ system. Long-range planned changes in NPRA processes could include a further reduction in maximum cycles. In addition, the total maximum duration for applicant responses could be limited to 6 months, with rejection of applications exceeding that limit.

### The Effect of Application Characteristics on Timing

In addition to the breakdown of timing, it is necessary to understand the effects of the qualities of individual applications such as priority designation, company type, or therapeutic area on approval timing. *Priority medicines:* Although the median approval time for all products approved in 2017 was 515 days, NPRA approved five priority medicines during that time, approximately 50% faster than those going through a standard review, clearly showing the potential for the agency to undertake a faster review. This expedited timing reflects the fact that NPRA reserves this designation for medicines that fulfil a vital unmet need for the people of Malaysia and both the agency and the applicant prioritize the dedication of resources to their authorization.

*Local versus international companies:* Local companies may not have the same resources to dedicate to the development of an adequate dossier or to respond to agency application deficiency questions in a timely manner, resulting in longer timelines for authorization. In this study, the median review time for the 20 product applications from international companies was 509 days compared with 557 days for six applications from local companies.

*Therapy area:* Additional agency and company resources may be dedicated to applications for products in traditional high-need therapeutic area such as anti-cancer, resulting in shorter timelines for authorization. In this study, the median review time for the 9 anti-cancer products was 481 days compared with 517 days for the other therapeutic areas.

### Moving Forward: Additional NPRA Improvements

In addition to planned changes in target timelines for scientific assessment initiation and maximum applicant response time and number of review cycles, NPRA is currently considering methods for overall improvements that can benefit both the agency and the applicant. First, the agency will continue its efforts to increase the speed of reviews, while balancing these efforts with the need to maintain patient safety. Second, NPRA will continue to improve staff efficiency by ensuring that they keep abreast with current developments and rapid advances in regulatory science. Third, NPRA work processes are currently being reorganized to improve the efficiency and to meet the needs of industry [16].

### Reliance and Cooperation

In order to achieve timely patient access to medicines, NPRA is moving toward greater emphasis on risk-based evaluations, focusing on what is locally critical and what can be leveraged or relied upon from the decisions made by stringent regulatory authorities through a reliance model. Two reliance routes, abbreviated and verification, are specified in the NPRA Guidelines on Facilitated Registration Pathways, which was made fully operational in April 2019 [17]. According to these guidelines, while the standard route has a target time of 365 days, the abbreviated route has a target timeline of 180 days and the verification route has a target of 135 days.

NPRA is also looking toward programs of cooperation and convergence to enhance and expedite its regulatory processes, providing ongoing regulatory training for WHO member states. NPRA has recently conducted bilateral technical meetings with regulatory agencies in Brunei, Japan, and Singapore and actively participating in three ASEAN working groups [10].

## Conclusions

The results of this study using OpERA methodology demonstrated the breakdown of the components of pharmaceutical regulatory timing in Malaysia into its components, that is, the time from receipt of data to start of scientific assessment, the scientific assessment (including both agency assessment and applicant response), and authorization of the product. The data for these individual components pointed to both challenges and their potential solutions.

In response to these data, the NPRA suggested three changes that could be made immediately: the implementation of a target goal of 100 days to initiate the scientific assessment of applications, the limitation of applicant correspondence to a maximum of five cycles, and the establishment of a total maximum duration for applicant responses 6 months. These initial study results will serve NPRA as a baseline for further assessment and comparison of NPRA efficiency after several planned improvements have been implemented.
